# New Protocol for Cell Culture to Obtain Mitotic Chromosomes in Fishes

**DOI:** 10.3390/mps1040047

**Published:** 2018-12-10

**Authors:** Fabilene G. Paim, Leandro Maia, Fernanda da Cruz Landim-Alvarenga, Fausto Foresti, Claudio Oliveira

**Affiliations:** 1 Department of Morphology, Sao Paulo State University, Botucatu 18618-689, Sao Paulo, Brazil; fforesti@ibb.unesp.br (F.F.); claudio.oliveira@unesp.br (C.O.); 2 Department of Animal Reproduction and Radiology, Sao Paulo State University, UNESP, Botucatu 18618-681, Sao Paulo, Brazil; leandromvet@hotmail.com (L.M.); fernanda.landim@unesp.br (F.d.C.L.-A.)

**Keywords:** cell culture, cryopreservation, fish cytogenetic, chromosomes, mitosis

## Abstract

Cell culture is an excellent alternative for the maintenance of cell lines and to obtain quality chromosome preparations of fishes. However, this methodology is still little employed, mainly because of the difficulty of standardization of cell cultures. In this study, we describe a methodology for the rapid acquisition of cell lineages and mitotic chromosomes for cytogenetic studies of fish species from muscle tissue cells. Our methodology is based on the use of a gelatin film, which provides better adhesion of a large number of cells and appropriate conditions for multiplication. The cells of *Astyanax altiparanae*, used as an experimental model, with fibroblast-like morphology, showed rapid cellular proliferation, resulting in a great number of cells. Chromosomal preparations of cultured cells showed the diploid number of the species, 2n = 50 chromosomes, in 80% of the cells examined, with chromosomes intact and distended. Cell populations were cryopreserved and after being recovered, these cells maintained their proliferative capacity. The development of this methodology represents an innovation for the fish cytogenetics area and it may bring a significant contribution to the conservation and study of several groups due to the difficulty of obtaining good-quality chromosome preparations.

## 1. Introduction

A large number of methods have been published for cell culture due to their enormous potential for application in different areas, such as biotechnology and aquaculture [1,2,3], toxicology [4,5], immunology [6,7], conservation [8,9,10] and cytogenetics [11]. Although cell culture has showed itself to be a great alternative for quality chromosome preparations, it is still little exploited with regard to obtaining chromosome preparations in Neotropical fish, mainly because of the difficulty of standardization of the isolation and maintenance of cell cultures.

In recent years, the development of new methodologies for fish cell cultures has increased following the development of the mammalian cell cultures. In general, these methodologies use a small tissue fragment to isolate the cells and, subsequently, to culture them in an adherent substratum [8,10,12,13], while other methodologies use proteolytic enzymes to isolate the cells of the tissue [14,15,16,17]. In addition, some of these methodologies have used artificial substrate to optimize and establish difficult cultures [15,16] (Table 1).

There are some advantages to using cell culture, such as: (1) In the cytogenetic study of small and large species, in which it is difficult to work with direct methods of chromosome preparation; (2) in the study species used in aquaculture or endangered species, when there is no possibility of sacrifice of animals; (3) in the testing of mutagenic or carcinogenic agents, without an unnecessary sacrifice of a large number of animals [12].

Another advantage of cell culture methodology is that the established cell banks are available at any time; thus, in case of repetition of cytogenetic methodologies, it is not necessary to go back to the field for new individuals. Considering the possible advantages of cell culture, the aim of this study was to develop a quick and easy methodology for fish cell culture, with a direct application, to obtain mitotic chromosomes for cytogenetic studies. The species *Astyanax altiparanae* (Characiformes) was used as a model, since this species has been the subject of different types of cytogenetic studies [18,19,20,21,22].

## 2. Materials and Methods

### 2.1. Preparation of Tissue and Isolation of Cells

Three individuals of *Astyanax altiparanae* (3 to 5 cm) from the Jararaca stream, a component of the Paranapanema River Basin, São Paulo, Brazil, were used to obtain the primary cell culture from samples of muscle tissue. The fishes were euthanized by immersion in ice water (−2 °C) [23] about five minutes and disinfected in 70% ethanol (*v/v*) for 1 min. The muscle tissues were removed aseptically using sterilized surgical equipment.

Voucher specimens were deposited in the fish collection of the Laboratory of Fish Biology and Genetics (LBP), UNESP, Botucatu (São Paulo State, Brazil). All experiments were conducted according to the Ethical Committee of Institute of Biosciences /UNESP/Botucatu, under protocol number CEUA-1001.

The tissue fragments were washed in 0.4% sodium hypochlorite solution (*v/v*) and 70% ethanol (*v/v*) for 30 s each, and then in Hank’s Balanced Salt Solution (HBSS, ThermoFisher Scientific, Waltham, MA, USA) with antibiotics (100 U/mL penicillin and 100 μg/mL streptomycin) (Thermofisher Scientific, Waltham, MA, USA) and antimycotic (2.5 μg/mL amphotericin B) (Thermofisher Scientific, Waltham, MA, USA) for 1 min. The tissue fragments were minced into small pieces (approximately 1 mm^3^ in size) in high glucose Dulbecco’s modified Egle’s medium (DMEM) (Thermofisher Scientific, Waltham, MA, USA) supplemented with antibiotics (100 U/mL penicillin and 100 μg/mL streptomycin) and antimycotic (2.5 μg/mL amphotericin). This medium was called the basal medium. The tissue fragments were centrifuged at 500× g for 10 min and, and the supernatants were discarded. This procedure was repeated one more time.

Then, 5 to 10 mL of a collagenase solution (0.4 mg/mL) (Millipore-Sigma, Burlington, MA, USA) was added to the tissue and kept in the incubator with orbital shaking at 27.5 °C and 90 rpm for a period of 30 to 90 min, according to the amount of tissue fragments. When the digestion was complete, the material was centrifuged and washed in basal medium for the removal of the collagenase solution.

Soon after, 10 to 20 mL of 0.25% trypsin solution (Thermofisher Scientific, Waltham, MA, USA) heated at 37 °C was added to the resulting pellet and maintained for 20 min at room temperature with successive pipetting. The supernatant resultant from this second digestion was collected and resuspended in complete medium (basal medium supplemented with 10% fetal bovine serum (FBS, Thermofisher Scientific, Waltham, MA, USA) and centrifuged at 500 g for 10 min.

### 2.2. Plate Treatment

The plates (12 multi-well plate; Sarstedt, Nümbrecht, Germany) were treated with 500 μL of 0.1% gelatin solution (Thermofisher Scientific, Waltham, MA, USA). Then, they were left in the incubator for approximately 2 h. Subsequently, the plates were removed from the incubator and the excess gelatin was removed with the aid of a pipette and only a gelatin film was kept.

### 2.3. Cell Culture and Subculture

The cells were inoculated into a 12-well plate and incubated at 27.5 °C in 5% CO_2_. The culture medium (basal medium supplemented with 10% FBS) was changed daily after adhesion and the plates were examined under an inverted microscope (LEICA DMI 4000B, Leica Microsystems, Wetzlar, Germany) to observe the cell growth. When the cells covered almost 80% of the plate (cell confluence), they were trypsinized with 0.25% trypsin solution, and then centrifuged and subcultured in plates (6 multi-well) and then in two flasks (T-25 cm^3^ Sarstedt, Germany), each of these multiplications called ‘passages’ here. This procedure was used to multiply the cells, using cells from one culture to create two new cultures and then successively repeating the procedure.

### 2.4. Cryopreservation and Thawing of Cells

Cells from the fourth to the sixth passage were used for cryopreservation. Cells were cultured in T-75 cm^3^ flasks (Sarstedt, Germany) and when they covered almost 80% of the flasks (confluency cell), they were trypsinized using the subculture protocol described above. The resulting pellet was resuspended in cryoprotectant medium 10% MERCK-SIGMA, (Darmstadt, Germany), 90% FBS, 1% antibiotics, 1% antimycotics) at a cell density of 10^5^ cells/mL and transferred to sterile cryovial (Sarstedt, Germany). The cryovials were placed into boxes filled with the proper amount of isopropyl alcohol (Nalgene, Rochester, NY, USA) and stored at −80 °C overnight for gradual freezing at 1 °C/min. After this period, the cryovials were removed from this support and then stored in ordinary boxes at −80 °C until the time of thawing.

For thawing, the cryovials containing cells were quickly thawed at 37 °C water bath. Then, the contents of the cryovials were added to a sterile falcon tube containing complete heated medium (basal medium supplemented with 10% FBS) and centrifuged. The pellet was suspended in complete medium and distributed in new vials and taken to the incubator under the same conditions as the initial culture. To estimate the number of viable cells and the cell concentration before and after thawing, the cells were counted in the hemocytometer (Neubauer camera) with trypan blue staining (Thermofisher Scientific, Waltham, MA, USA).

### 2.5. Chromosome Preparations

A flask (T-25 cm^3^ Sarstedt, Germany) was used for cytogenetic analysis. The muscle cells from the third to the sixth passage were treated with 100 μL of 0.0016% colchicine solution, which was added to the flask for up to 4 h. Subsequently, the culture cells were trypsinized and centrifuged at 500 g for 10 min. The cells were treated with 0.075 M potassium chloride solution (KCL) for 15 min at 37 °C and fixed with Carnoy’s solution (3 methanol:1 acetic acid). Then, this cell suspension was washed twice with Carnoy’s solution. The cell suspension was deposited on slides and stained with Giemsa 5% for 8 min to observe the chromosomes.

## 3. Results and Discussion

**Figure 1:**
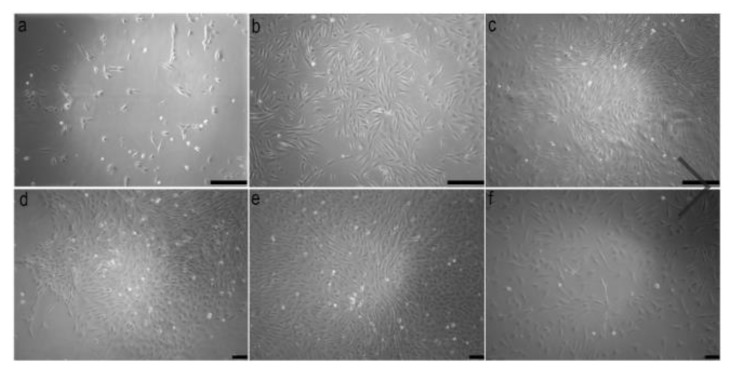
Morphology of attached cell from enzymatic digestion of the muscle tissues of *Astyanax altiparanae* after three days (**a**). These cells showed more than 80% of cell confluence after 10 days (**b**) and when subcultured, the cell confluence was between two and three days. Note the cells at third passage after three days (**c**). In (**d**), a colony of fibroblastic cells is shown in the fourth passage six days after thawing. These cells show cell confluence after 18 days (**e**) and when subcultured, these cells cover the bottom of the flask in four days. Cell after thawing in the sixth passage after two days of being subcultured. Scale bars 200 µm in (**a**–**c**); 100 µm in (**d**–**f**) (10×).

The cells isolated from the muscle tissue of *Astyanax altiparanae* showed adhesion to the bottom of the flask between 24 and 48 h and they started to acquire fibroblastic morphology after 72 h (Figure 1a). The isolation protocol used in the present study with the aid of proteolytic enzymes (collagenase and trypsin) was demonstrated to be efficient in previous studies in isolating cells from skin and fin tissues [11,15], heart [16,24], brain [17], and gonads [25].

The isolated cells showed rapid growth, achieving cellular confluence in primary culture up to 10 days and when they were subcultured between 2–3 days (Figure 1b,c). This rapid growth was attributed to the use of gelatin-based film, which allowed the adhesion of a large number of cells and provided suitable conditions for cell multiplication. Cell populations need adhesive substrates for proliferation and differentiation, according to Alexander et al. [26], Graf et al. [15], and Kim et al. [16], among others, as observed here. Thus, the use of gelatin film can be considered an excellent adhesive material for cell proliferation due to its biological origin derived from the collagen and its constitution of amino acids, mainly proline and hydroxyproline [27]. According to Kim et al. [16], the adhesion of a high density of cells to the substrate will allow a cell–cell contact, ensuring the cellular survival, proliferation, and differentiation in the culture.

Prior to the application of this gelatin film to the cell culture, one of the difficulties for standardization of the cell culture protocol in fish was the poor adhesion of the cells to the substrate, which led to a possible inhibition of the proliferation. The use of 0.1% gelatin on the plate treatment allowed, in addition to better adhesion, interactions among the cells, providing a quick and appropriate growth. In fact, cell adhesion is essential for tissue development and maintenance, with the exception of hematopoietic cells; all cells derived from tissues are dependent on a substrate for their anchorage [28,29].

Additionally, another important aspect in the protocol proposed here was the daily medium change, allowing the renovation of the nutrients necessary for cell growth. The culture medium is one of the most important factors in animal cell culture, and its function is providing adequate nutrients, pH, and osmolarity for the survival and multiplication of cells [30]. In this study, the medium used was high glucose DMEM supplemented with 10% fetal bovine serum, which was used by Graf et al. [15] and Wang et al. [10] to establish fish cell lines. Although some fish cell cultures use the L-15 medium [17,24], our study showed that high glucose DMEM is more satisfactory for cell growth in *A. altiparanae*.

The viability of the cells of *A. altiparanae* before cryopreservation was 98% and the recovery was up to 71% after thawing. These cells were stable and able to survive in low-temperature storage for more than a year. It is known that all cryoprotectants may have some negative effect on the cells, since the percentage of viability and the adhesion of thawed cells may be reduced by the toxicity of cryoprotectant [10,31,32]. Dimethyl sulfoxide (DMSO) is the most common cryoprotectant used in the fish cell culture [32,33], with a concentration of either 5 or 10%. The latter was the concentration used for the cryopreservation of the samples in our study and worked very well according to our results.

Cells cultured after thawing acquired a fibroblast-like morphology up to three days and, progressively, some colonies could be observed after six days of being cultured (Figure 1d). These cells showed cell confluence after 18 days (Figure 1e) and covered the bottom of the flask up to four days when subcultured (Figure 1f). Cells from cryopreservation have a slower proliferation capacity after thawing, since these cells need to survive the stress of freezing and still adapt to new culture conditions [30].

**Figure 2:**
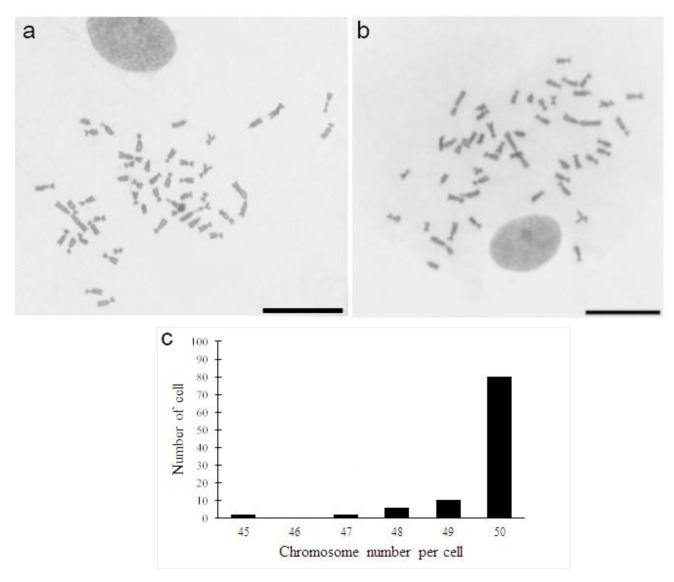
Chromosomes in metaphases obtained from fibroblastic cells of *Astyanax altiparanae* in the fourth passage before cryopreservation (**a**) and sixth passage after thawing (**b**). Scale bars 20 µm. Frequency distribution of chromosomes of cells of *Astyanax altiparanae* in cells spreads (**c**).

A cytogenetic analysis of cell cultures obtained from *A. altiparanae* revealed a diploid modal number of 2n = 50 chromosomes before and after thawing (Figure 2a,b) in 80% of the cells analyzed (Figure 2c). Similar results, in our research group (unpublished data), were obtained using the technique of Foresti et al. [34], a fact that reinforces our results under these experimental conditions. It is known that cell stress conditions (e.g., freezing and thawing of cells) or even subcultures may lead to chromosomal aberrations, such as changes in morphology and chromosome numbers [35], which was observed in low amounts in the metaphase cells of *A. altiparanae* until the 7th passages, when these cells were cryopreserved again. This methodology opens up new opportunities in the area of fish cytogenetics.

Since the chromosomes obtained by culture are more sensitive to hypotonic treatment, different periods of exposures to the hypotonic solution (KCL 0.075 M) were tested in order to improve the quality of the chromosome preparations related to the dispersion and morphology of the chromosomes. Thus, the results obtained with the 15 min hypotonic treatment were the most adequate for the cell culture of this species, within an interval of 10 to 30 min.

The success of the cell culture technique also depends on the biology of the species or group of organisms in study [35]. Thus, the temperature and incubation conditions of the cells are dependent on the animal body temperature from which the cells were isolated. For this experiment, it was verified that the temperature of 27.5 °C and incubation conditions with 5% of CO_2_ were the most suitable conditions.

## 4. Conclusions

The positive results of the methodology to isolate and cultivate the fibroblastoid cells of *A. altiparanae* using enzymatic digestion followed by cell culture using a gelatin-based film are a great innovation in the area of fish cytogenetics and conservation. This methodology was applied successfully in other species of Characiformes (*Hyphessobrycon*, *Colossoma*, *Mimagoniatus*, *Piaractus*, and *Megaleporinus*). The application of this procedure can result in a significant contribution in studies of fish groups, few of which have been explored cytogenetically due to the difficulty in obtaining good quality chromosome preparations which are crucial for the success of several experiments.

## Figures and Tables

**Table 1:** Main techniques used for fish cell cultures and their conditions of cultures.

Species	Tissue	Methods for Isolate Cells	Type of Medium	% FBS ^1^	Temperature (°C)	Plate Treatment	Reference
*Epinephelus malabaricus*	Gill	Mechanical methods (Explants)	L-15 ^2^	20%	28° ± 2 °C	No	[8]
*Anabarilus graham*	Fin	Mechanical methods (Explants)	DMEM ^3^/F-12	20%	28 °C	No	[10]
*Notropis* ssp.	Fin and scales	Mechanical methods (Explants)	199	10%	30 °C	No	[12]
*Sparus aurata* L.	Fin	Mechanical methods (Explants)	DMEM ^3^/F-12	15%	15%	No	[13]
*Thunnus maccoyii*	Fin, muscle, skin and spinal	Proteolytic enzymes (collagenase)	L-15 ^2^	15–20%	25 °C	No	[14]
*Nothobranchius furzeri*	Skin and fin	Proteolytic enzymes (collagenase)	DMEM^3^	10%	28 °C	Yes (Gelatin)	[15]
*Acipenser baerii*	Heart	Proteolytic enzymes (collagenase/trysin-EDTA)	L-15 ^2^ and DMEM ^3^	20%	28 °C	Yes (Gelatin)	[16]
*Siniperca chuatsi*	Bain	Proteolytic enzymes (collagenase)	L-15 ^2^	20%	28 °C	No	[17]
*Astyanax altiparanae*	Muscle	Proteolytic enzymes (collagenase/trysin-ETDA)	DMEM ^3^	10%	28 °C	Yes (Gelatin)	Present study

^1^ Fetal bovine serum (FBS); ^2^ Leibovitz L-15; ^3^ Dulbecco’s modified Egle’s medium (DMEM).
